# Frequency specific contribution of intrinsic connectivity networks to the integration in brain networks

**DOI:** 10.1038/s41598-019-40699-z

**Published:** 2019-03-11

**Authors:** Yeong-Hun Park, Jungho Cha, Viktoriya Bourakova, Jong-Min Lee

**Affiliations:** 1 Department of Biomedical Engineering, Hanyang University, Seoul, Korea; 2 Memory and Aging Center, Department of Neurology, University of California, San Francisco, San Francisco, CA USA

## Abstract

Brain networks are integrated and segregated into several intrinsic connectivity networks (ICNs). Frequency specificity of ICNs have been studied to show that different ICNs have a unqiue contribution to brain network integration along frequencies. The purpose of this study was to evaluate the contribution of individual ICN to brain network integration along their frequency. We used 14 ICNs and determined 2 frequency bands (LF1, 0.03~0.08 Hz and LF2, 0.009~0.012 Hz) from the hierarchical clustering of 101 frequency bins. We proposed a novel measure, called ICN efficiency, representing the difference between the global efficiencies of the whole brain network with and without the ICN to evaluate the contribution of the ICN to brain network integration. We found that each ICN had a different ICN efficiency at 2 frequency bands. We also found that the distinct subregions of the same ICN had a frequency specific contribution to brain network integration. Futhermore, the integration with other ICNs of the distinct subregions of the same ICN were different at 2 frequency bands. In conclusion, the contribution of each ICN to brain network integration is frequency specific and distinct subregions of the same ICN have functionally distinct roles with other ICNs at 2 frequency bands.

## Introduction

Functional connectivity (FC) is defined as the correlation of low frequency (0.009~0.08 Hz) fluctuations among anatomically distinct brain areas on resting-state functional magnetic resonance imaging (fMRI)^1^. FC has been widely used to gain insight into the fundamental functional architecture of the brain^2^ and to identify intrinsic connectivity networks (ICNs), which are determined by spatially independent and temporally correlated FC^3^. There have been reported 10~14 ICNs associated with intrinsic functions such as vision, hearing, language, working memory, visuospatial attention, salience processing and episodic memory in the resting-state brain^4–10^.

Several studies revealed specific frequencies for ICNs within the low frequency spectrum of blood oxygen level dependent (BOLD) signal and suggested that different ICNs likely have unique intrinsic frequencies^11–15^. Mantini, *et al*.^15^ showed that 6 ICNs that fluctuate at slightly different BOLD signal frequencies were correlated with specific electrophysiological rhythms. Wu, *et al*.^14^ showed that cortical networks concentrate within ultra-low frequency range (0.01~0.06 Hz), while connections within limbic networks distribute over a wider frequency range (0.01~0.14 Hz). Sasai, *et al*.^12^ and Thompson and Fransson^13^ showed the frequency specificity of ICNs from the viewpoint of whole brain network integration and segregation. Chen, *et al*.^11^ demonstrated that ICN information of the slow-4 (0.027~0.073 Hz) and slow-5 (0.01~0.027 Hz) frequency bands are complementary in classifying between autism spectrum disorder and healthy controls. Among the low frequency divisions of previous studies (Table 1), the most used frequency bands are the slow-4 and slow-5 frequency bands of Zuo, *et al*.^16^. The criteria for slow-4 and slow-5 frequency bands were based on electro-physiological data from rats^17^. Observing the frequency specific contribution of ICN is an analysis in terms of the connectivity network, so it may be more appropriate to determine the frequency band based on connectivity matrix constructed using the BOLD signal rather than based on the electro-physiological frequency bands.

**Table 1:** The low frequency division in previous studies.

Previous studies	Frequency
Wu *et al*.^14^	Ultra-low frequency (0.01–0.06 Hz), Wider frequency (0.01–0.14 Hz)
Zuo *et al*.^16^	Slow-5 (0.01–0.027 Hz), Slow-4 (0.027–0.073 Hz)
Baria *et al*.^57^	LF (0.01–0.05 Hz), MF1 (0.05–0.10 Hz), MF2 (0.10–0.15 Hz), HF (0.15–0.20 Hz)
Sasai *et al*.^12^	Very low frequency band (0.01–0.03 Hz), Low frequency band (0.07–0.09 Hz)
Qian *et al*.^58^	IMF5 (0–0.015 Hz), IMF4 (0.01–0.025 Hz), IMF3 (0.025–0.05 Hz), IMF2 (0.05–0.11 Hz), IMF1 (0.11–0.22 Hz)
Thompson and Fransson^13^	F1 (0.016 Hz), F2 (0.028–0.037 Hz), F3 (0.071–0.08 Hz)

Brain networks combine to form one integrative complex network linking all brain regions and multiple ICNs together into one complex system^18^. Global and nodal efficiencies have been widely adopted to observe the integration of the brain network as graphical measures based on the shortest path length^19,20^. Sasai, *et al*.^12^ evaluated the integration of a brain network composed of 3 ICNs using global efficiency and detected hubs by considering the nodal degree and eigenvector centrality. However, global and nodal efficiencies are insufficient to evaluate the contribution of each ICN to brain network integration because they are indicators for the overall network or each node. Also, since the definition of functional integration is commonly based on the concept of a path^21^, the hub defined by nodal degree and eigenvector centrality are insufficient to evaluate functional integration than the hub defined by path-based measures. Unlike Sasai, *et al*., Thompson and Fransson^13^ evaluated the contribution of an ICN to the integration of a brain network composed of 10 ICNs using the strength contribution and detected hubs by considering the betweenness centrality (BC) based on the concept of a path. However, this may also not be sufficient for evaluating the contribution of an ICN to the integration of the brain network because it is based on each edge’s value over the sum of all edges and only considers the first degree of each node.

Separating an ICN from the whole brain network and computing its efficiency could not evaluate the contribution of the ICN in brain network integration properly. A general approach to measure the efficiency of a specific node is to calculate the inverse of the average shortest path length between the first neighbors of the node in the condition while excluding the node^19^. This approach is known as local efficiency and indicates how efficiently the node’s first neighbor communicates when the node is removed. We applied the same concept to measure the efficiency of the ICNs and proposed a novel measure, called ICN efficiency (E_ICN_), defined as the difference between the global efficiency and the efficiency of the whole brain network excluding the ICN. This could result in evaluating the contribution of an ICN to whole brain network integration.

The purpose of this study was to evaluate the contribution of an ICN to brain network integration using E_ICN_ along the frequency. First, the connectivity matrices along the frequency band were constructed from time-varying frequencies of the brain network consisting of 14 ICNs. Hierarchical clustering analysis was conducted to choose 2 frequency bands. E_ICN_ was calculated at each frequency band.

## Results

### Dividing the low frequency range into the frequency bands

**Figure 1:**
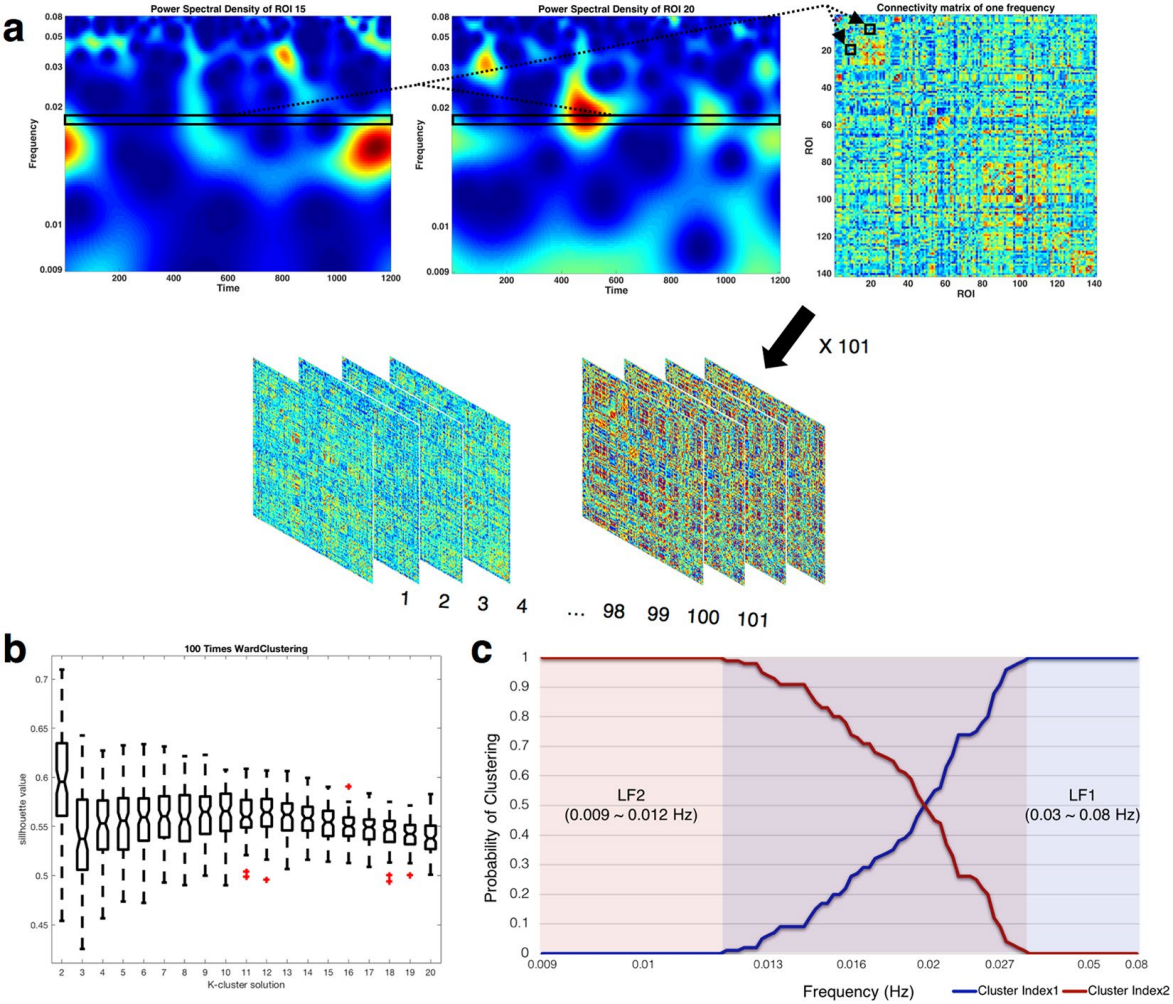
Construction of the connectivity matrix and division frequency bands. (**a**) A connectivity matrix was constructed for each frequency bin by using Pearson’s correlation coefficient of each power spectral density (PSD) time-course through all nodes. The black rectangular small box indicates PSD time-course of specific frequencies. A 141 × 141 connectivity matrix of specific frequencies with a total of 101 frequency bins is presented. (**b**) The hierarchical clustering between group averaged connectivity matrices of each frequency was calculated by using Jensen Shannon divergence. For the highest silhouette value, 101 frequency matrices were clustered into 2 subbands. (**c**) Probability of clustering index for each frequency bin. Since the probability of the intermediate frequency band is uncertain, only the frequency bands at both ends were defined as the final 2 subbands (LF1: 0.03–0.08 Hz, LF2: 0.009–0.012 Hz).

We divided the low frequency range into 101 bins to construct connectivity matrix at each bin. The average silhouette values for ward clustering were obtained as a function of k. The number of clusters were determined as 2 because the silhouette value was the highest when using k = 2 (Fig. 1b). (See Supplementary Fig. S1 and Table S1 for the silhouette value about the number of clustering iteration). The resulting frequency bands were LF1 (0.03~0.08 Hz) and LF2 (0.009~0.012 Hz) (Fig. 1c).

### Changes of E_ICN_ along the frequency bands

**Figure 2:**
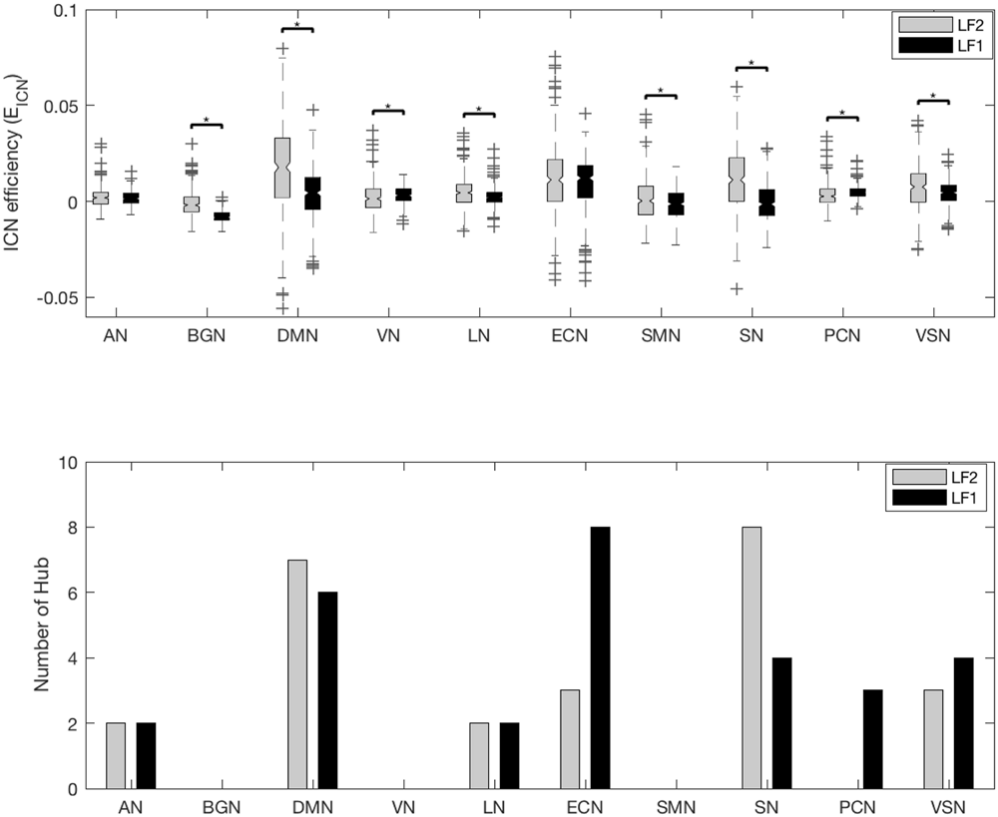
E_ICN_ and number of BC hubs along the frequency band. The E_ICN_ of the 10 ICNs was compared using a paired sample t-test analysis between the 2 frequency bands. The results were corrected by Bonferroni correction (p < 0.005). The tendency of E_ICN_ and number of BC hubs along the frequency bands were different for each ICN. The tendencies of E_ICN_ and number of BC hub along the frequency bands were similar in AN, DMN, SN and PCN. The abbreviations used were as follows: auditory network (AN), basal ganglia network (BGN), default mode network (DMN), visual network (VN), language network (LN), executive control network (ECN), sensorimotor network (SMN), salience network (SN), precuneus network (PCN), visuospatial network (VSN).

E_ICN_ was calculated to determine whether the contribution of ICNs to brain network integration differs along the frequency bands (Fig. 2 shows significant differences in t-values with Bonferroni correction (p < 0.005)). The number of BC hubs were plotted as the indicator of E_ICN_ together. Note that the statistically significant difference in E_ICN_ and the difference in the number of BC hubs were consistent in the Auditory network (AN), Default mode network (DMN), Salience network (SN) and Precuneus network (PCN). Most ICNs had a positive E_ICN_ value except that basal ganglia network (BGN) had a negative E_ICN_ value. There are 3 distinct groups according to the pattern of E_ICN_ values along the frequency bands; (1) higher at LF2 than at LF1: DMN, Language network (LN), Sensorimotor network (SMN), SN and Visuospatial network (VSN), (2) lower at LF2 than at LF1: BGN, Visual network (VN) and PCN, (3) similar at LF1 and LF2: AN and executive control network (ECN). These results suggest that the contribution of each ICN to brain network integration is frequency specific.

### Different integration between distinct subregions of the same ICN along the frequency band

**Figure 3:**
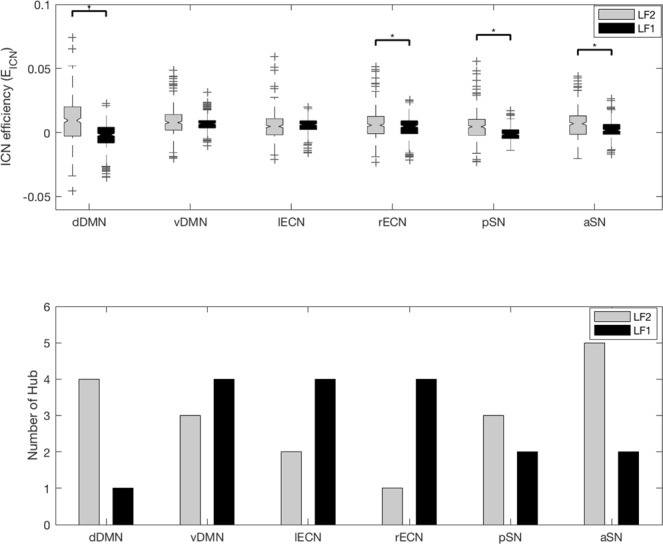
E_ICN_ and number of BC hubs along the frequency band. The E_ICN_ of the 6 sub-ICNs was compared using a paired sample t-test analysis between the 2 frequency bands. The results were corrected by Bonferroni correction (p < 0.008). The tendency of E_ICN_ and number of BC hubs along the frequency bands were different for each ICN. The tendencies of E_ICN_ and number of BC hub along the frequency bands were similar in dDMN, pSN and aSN. The abbreviations used were as follows: dorsal default mode network (dDMN), ventral default mode network (vDMN), left executive control network (lECN), right executive control network (rECN), posterior salience network (pSN) and anterior salience network (aSN).

Three well-known ICNs that can be divided into distinct subregions were determined and E_ICN_ was calculated along the frequency bands with 6 sub-ICNs: dorsal DMN (dDMN), ventral DMN (vDMN), left ECN (lECN), right ECN (rECN), posterior SN (pSN) and anterior SN (aSN) (Fig. 3 shows significant differences in t-values with Bonferroni correction (p < 0.008)). Note that the statistically significant difference in E_ICN_ and the difference in the number of BC hubs were consistent in dDMN, pSN and aSN. The dDMN showed greater E_ICN_ at LF2, but the vDMN showed a similar E_ICN_. The rECN showed greater E_ICN_ at LF2, but the lECN showed a similar E_ICN_. The pSN and aSN showed greater E_ICN_ at LF2. These findings show that the distinct subregions of the same ICN had a frequency specific contribution to integration of brain network.

**Figure 4:**
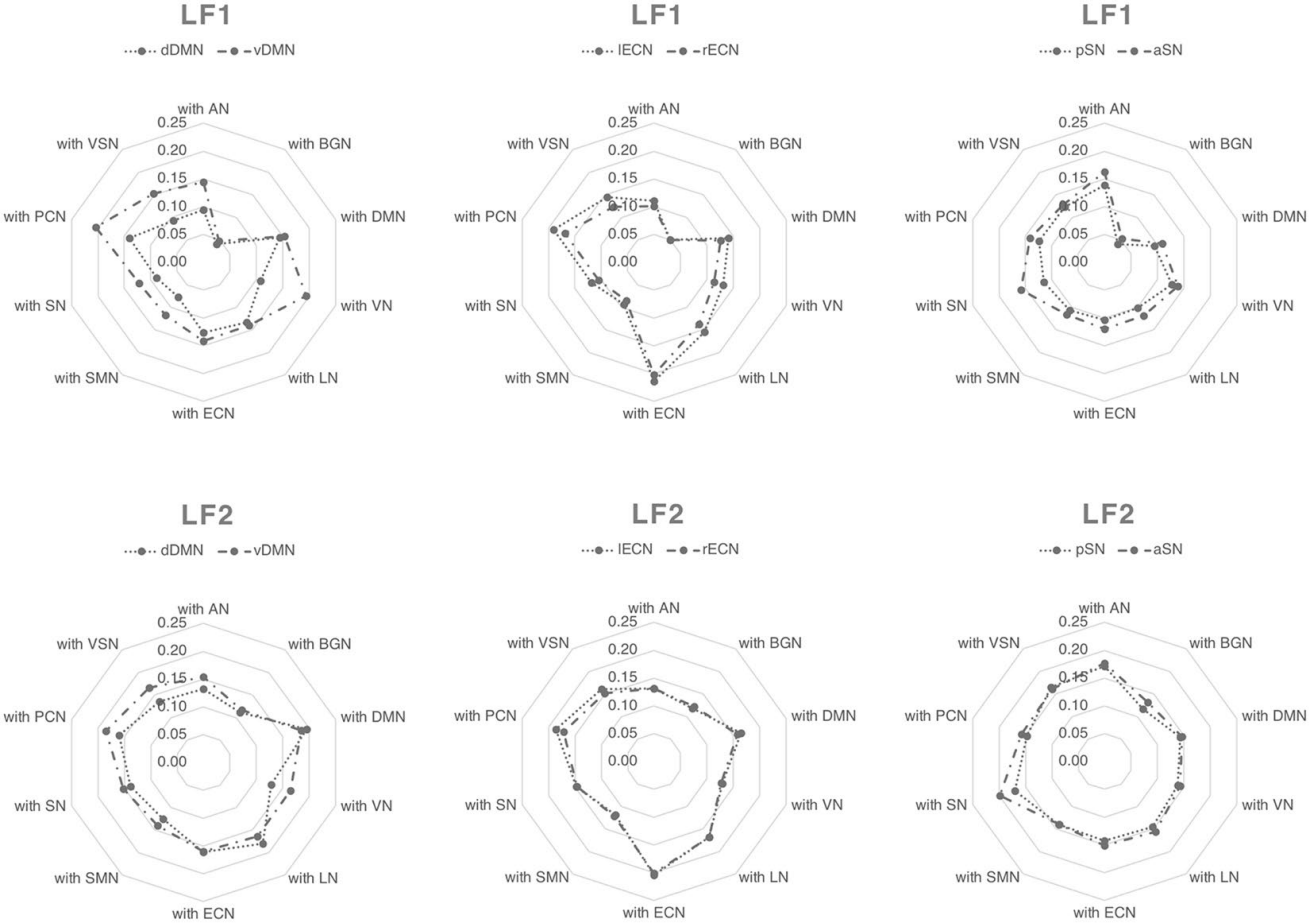
E_ICN-ICN_ between sub-ICN and other ICN along the frequency band. The E_ICN-ICN_ were calculated at LF1 and LF2. The patterns of E_ICN-ICN_ were mainly different at LF1. The results were corrected by Bonferroni correction (p < 0.0025). The abbreviations used were as follows: dorsal default mode network (dDMN), ventral default mode network (vDMN), left executive control network (lECN), right executive control network (rECN), posterior salience network (pSN), anterior salience network (aSN) auditory network (AN), basal ganglia network (BGN), visual network (VN), language network (LN), sensorimotor network (SMN), precuneus network (PCN), visuospatial network (VSN).

In addition, the efficiency between each sub-ICN and the other ICNs (E_ICN-ICN_) was calculated along the frequency bands (Fig. 4). Comparing each sub-ICN pair, the vDMN showed a greater E_ICN-ICN_ with other ICNs except for DMN and LN than dDMN at LF1. However, the vDMN showed a greater E_ICN-ICN_ with AN, VN, SN, PCN and VSN than dDMN at LF2. The lECN showed a greater E_ICN-ICN_ with DMN, VN, LN, SN,PCN and VSN than rECN at LF1. However, the lECN showed not difference in E_ICN-ICN_ with all other ICNs compared from rECN at LF2. The aSN showed a greater E_ICN-ICN_ with AN, BGN, DMN, LN, ECN, SN and PCN than pSN at LF1. However, the aSN showed a greater E_ICN-ICN_ with SN than pSN at LF2. The difference in results was corrected with Bonferroni correction (p < 0.0025). The results suggested that the efficiency of each sub-ICN pair with the other ICNs is more different at LF1 than at LF2.

### Consistent tendency of E_ICN_ to frequency of other data sets

**Figure 5:**
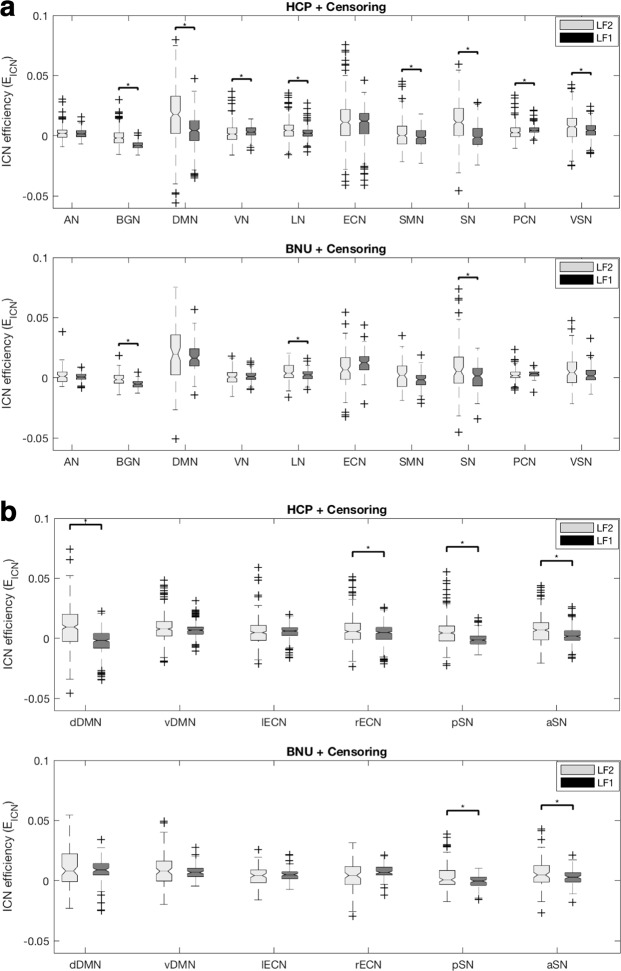
Impact of the data set on E_ICN_ by frequency. (**a**) Difference of E_ICN_ by frequency according to the data set. The E_ICN_ result of BNU data differ from the E_ICN_ results of HCP data in statistical significance of DMN, VN, SMN, PCN, and VSN, but the tendency of E_ICN_ to frequency is maintained. (**b**) Difference of E_ICN_ of 6 sub-ICN by frequency according to the data set. The statistical tendency of sub-ICN not reverse. The abbreviations used were as follows: dorsal default mode network (dDMN), ventral default mode network (vDMN), left executive control network (lECN), right executive control network (rECN), posterior salience network (pSN), anterior salience network (aSN), auditory network (AN), basal ganglia network (BGN), visual network (VN), language network (LN), sensorimotor network (SMN), precuneus network (PCN), visuospatial network (VSN).

The E_ICN_ was calculated in two data sets to prove that the results of E_ICN_ are not data set dependent. When the E_ICN_ results of Human Connectome Project (HCP) S900 data and Beijing Normal University (BNU) data were compared, the results of BNU data differed from the results of HCP data in statistical significance of DMN, VN, SMN, PCN and VSN, but the tendency of E_ICN_ to frequency was maintained (Fig. 5a). Although there was a statistical difference, the tendency of E_ICN_ to frequency of BNU data was the same as the tendency of E_ICN_ to frequency of HCP data. This result indicates that ICN’s frequency-specific contribution to the integration in brain networks is robust. In addition, when the 6 sub-ICN efficiency results of HCP data and BNU data were compared, the statistical tendency of sub-ICN did not reverse (Fig. 5b).

## Discussion

In this study, we showed that ICNs make frequency specific contributions to the integration within the brain network (Fig. 2) and that the distinct subregions of the same ICN showed different characteristics along the frequency bands (Figs 3 and 4).

We determined 2 frequency bands (LF1, 0.03~0.08 Hz and LF2, 0.009~0.012 Hz) by hierarchical clustering which considered the FC matrix based on the BOLD signal. The FC frequency bands were slightly different from the electro-physiological frequency bands (slow-4, 0.027~0.073 Hz and slow-5, 0.01~0.027 Hz). The slow-4 range was included in LF1 and the slow-5 range was overlapped with LF2. Zuo, *et al*.^16^ showed that slow-4 fluctuations are higher in the subcortical regions and several sensorimotor regions than slow-5 and slow-4 fluctuations are lower in the ventromedial regions than slow-5. Since then, many clinical studies of neurological and psychiatric disease^11,22–24^ have used slow-4 and slow-5 to reveal regions of different brain activity from normal controls. Although many significant results were observed using slow-4 and slow-5, the slow-frequency bands are pre-defined frequency bands based on electro-physiological data from rats^17^, as mentioned earlier. Therefore, it was necessary to find the frequency band by considering the FC matrix, and the LF1 and LF2 we obtained could be considered as fine-tuned frequency bands from slow-4 and slow-5 frequency bands. In future studies, it is better to use the fine-tuned frequency bands LF1 and LF2 than to use the slow-4 and slow-5 frequency bands when analyzing frequency-specific brain networks rather than analyzing frequency-specific brain activity measured by amplitude of low frequency fluctuation.

We proposed E_ICN_ as a novel network measure for evaluating the contribution to brain network integration. While the global efficiency represents the integration of the whole brain and strength contribution considers the first degree neighbors only^12,13^, E_ICN_ is a path-based method which considers whole brain network nodes as a quantitative indicator of the contribution of ICN to the integration of the whole brain network. The fact that E_ICN_ changes along frequency bands illustrates how the contribution of an ICN to brain network integration differs across frequency (Fig. 2). Note that each ICN had a different pattern of E_ICN_ values along frequency, which is similar to the frequency characteristics of each ICN observed through strength contribution in another study^13^. E_ICN_ of BGN had a negative result at all frequencies. Although BGN is known to have FC with other regions of the cortex^25^, the results indicate that BGN interferes with brain network integration, therefore, more research about the contribution of BGN to brain network integration is needed.

We examined E_ICN_ with sub-ICN of three ICN along frequency bands to investigate different integrations between distinct subregions of the same ICN along the frequency band (Fig. 3). We also calculated E_ICN-ICN_ with sub-ICN of three ICN to investigate different integrations with other ICNs (Fig. 4). A central component of the DMN resides in the posterior cingulate cortex (PCC), which is functionally correlated and separated into dorsal and ventral areas by cytology^26^. The dorsal PCC is strongly functionally connected with the DMN and with the cognitive control network (CCN), which is typically involved in the control of externally directed behavior, while the ventral PCC is more strongly integrated with the DMN^27–29^. In our study, the dDMN and vDMN had a different E_ICN_ along the frequency band. The vDMN showed a larger E_ICN-ICN_ with other ICNs than dDMN at LF1. This suggests that the vDMN integrates with other ICNs more than dDMN at LF1. To the best of our knowledge, this study is the first to demonstrate that distinct subregions of DMN play functionally distinct roles with the other ICNs in the frequency domain. The lECN and rECN showed a different E_ICN_ along the frequency band in spite of having the same region of interest (ROI) in the corresponding hemisphere. The lECN and rECN are known to associate different temperaments in motivation^30^, which is probably related to the observation that the lECN showed a larger integration with other ICNs than rECN at LF1. The SN is divided into the aSN and pSN by structural and functional connectivity with the anterior and posterior insula^31,32^. The aSN and pSN had the same number of BC hubs at LF1, but the aSN showed a larger integration with other ICNs than pSN at LF1. The aSN plays a critical role in switching between the ECN and DMN^33^, which may be one of the explanations for the aSN showing a larger E_ICN-ICN_ with the DMN and ECN than pSN at LF1. From these observations we confirm that the DMN and ECN are integrated centering on the aSN at LF1, indicating the frequency specificity of ICN to the integration in the brain network.

The present study has several limitations. First limitation is that different pre-processing methods lead to slightly different results (See Supplementary Fig. S2). The E_ICN_ results of three pre-processing pipelines differed from each other in statistical significance, but the tendency of E_ICN_ to frequency was maintained. These results indicate that the censoring step and global signal regression (GSR) step do not necessarily impact the magnitude of E_ICN_ along the frequency. In the end, we decided to include the preprocessing step by determining whether it was necessary or not, rather than judging whether the result was right or wrong. Therefore, we included the censoring step because it was necessary to exclude the large motion effect from the BOLD signal. However, the GSR step was not included because it was assumed that the nuisance-signal was already sufficiently removed in the ICA-fixed step. Second limitation is selection of hyperparmeters for the Morlet wavelet transform. An angular frequency *ω*_0_ = 6, known to provide a good tradeoff between time and frequency localization, was set as in the previous^13,34–36^. For other hyperparameters, there was no information to set by reference. Nevertheless, our purpose was not to compare each frequency bin, but to compare the frequencies after clustering them into subbands, so we have compared clustering results under various conditions (See Supplementary Fig. S3). When the frequency clustering results are compared by changing the smallest scale ‘*s*_0_’ and spacing between scales ‘*ds*’, cluster colution 2 had the highest silhouette value under any conditions.

## Methods

### fMRI data

The present study used HCP S900 data (http://humanconnectome.org/) released in December 2015^37^. In the HCP S900, 352 subjects (178 females and 173 males, age 22–36) were selected after excluding twin subjects for further analysis. The experiments were performed in accordance with relevant guidelines and regulations and all experimental protocol was approved by the Institutional Review Board (IRB) (IRB # 201204036; Title: ‘Mapping the Human Connectome: Structure, Function, and Heritability’). Written informed consent was obtained from all participants. Our data analysis was performed in accordance with ethical guidelines of the Hanyang University ethics committee. Data were collected on a customized Siemens 3 T Connectome Skyra magnetic resonance imaging (MRI) with a standard 32-channel head coil at the Washington University in St. Louis. Echo planar imaging (EPI) time-series scans consisted of 1200 volumes and lasted for approximately 15 minutes with the following imaging parameters: TR = 720 ms; TE = 33.1 ms; flip angle = 52°; field of view = 208 × 180 mm^2^; matrix size = 104 × 90; a multiband factor of 8; 72 slices; with voxel dimension = 2 × 2 × 2 mm^3^. Participants were instructed to rest with their eyes open with relaxed fixation on a projected bright cross hair on a dark background during each scan. We used ‘ICA-FIX cleaned’ resting-state fMRI data only, which was preprocessed for co-registration, normalization, head motion correction, and artifact rejection^38,39^. The ICA-FIX cleanup preprocessing step regressed out non-neural contributions from fMRI data. The non-neural contributions are motion-related timecourses and artefact components identified using independent component analysis (ICA) with a new FSL tool FIX (FMRIB’s ICA-based X-noisfier)^40^. For the additional pre-processing step, volume censoring was applied by using the frame-to-frame displacement (FD) method^41^. If the FDs exceeded a threshold of 0.35 at a time point, the time points values were censored and replaced by interpolated neighboring non-censored values. 51% of the total data included the censoring volume, with an average 13.2 out of 1200 volumes (1.1% of the fMRI data per subject). Among them, the censored volume ratio of two data were 25% and 35.91% respectively, and the analysis was carried out with a total of 350 data excluding the two data. We didn’t include the GSR step because the ‘ICA-FIX cleaned’ data was already sufficiently regressed out the nuisance signals and the GSR step had a controversy^42^. We repeated the E_ICN_ using the State Key Laboratory of Cognitive Neuroscience and Learning at BNU  (n = 100), which is part of the 1,000 Functional Connectomes Project (http://fcon_1000.projects.nitrc.org/)^43^. The BNU EPI time-series scans consisted of 225 volumes and lasted for 7.5 minutes with the following imaging parameters: TR = 2000 ms; 33 slices; with voxel dimension = 3.125 × 3.125 × 3.6 mm^3^. The fMRI data pre-processing proceeded according to our previous study^28,44–48^. The pre-processing included despiking, slice-timing, head motion correction, ANATICOR method^49^ and volume censoring.

### Constructing the nodes of the brain network

We selected 141 functional ROIs as nodes covering 14 ICNs consisting of the AN, BGN, dDMN, vDMN, higher VN (hVN), primary VN (pVN), LN, lECN, rECN, SMN, aSN, pSN, PCN and VSN^9^ from 499 functional ROIs originally defined by Richiardi, *et al*.^50^.

### Constructing the connectivity matrix

We decomposed the averaged preprocessed time-series within each ROI into the time-frequency domain by convolving them with the complex Morlet wavelet function^51^ and obtained power spectral density (PSD) maps by calculating the square of the magnitude of the continuous wavelet transform coefficient across time. For the Morlet wavelet, the hyperparmeters were used as follows: angular frequency *ω*_0_ = 6, smallest scale *s*_0_ = 1, spacing between scales *ds* = 1, and number of scales *NbSc* = 128. We generated 101 frequency bins with a frequency resolution of roughly 0.001 Hz in the low frequency range (0.009~0.08 Hz) and constructed a connectivity matrix for each frequency bin using the Pearson’s correlation coefficient among the PSD time-courses of the nodes (Fig. 1a).

### Dividing the low frequency range into frequency bands

We randomly selected 100 subjects from the 350 HCP subjects and constructed a group connectivity matrix with 101 frequency bins. The differences among the group-averaged connectivity matrices of each frequency bin were calculated using Jensen Shannon divergence^52,53^. The hierarchical clustering of connectivity matrices along the frequency band was performed to cluster the 101 frequency bins. We repeated the above procedure 100 times. The average silhouette values for ward clustering were obtained as a function of k (Fig. 1b). Since the silhouette value was highest when using k = 2, the frequency bins were clustered into 2 frequency bands. The probability of the clustering index at each frequency bin was calculated to decide robust frequency range (Fig. 1c). Since the probability of the intermediate frequency band is uncertain, we defined both end frequency bands as the final 2 frequency bands: LF1, 0.03~0.08 Hz and LF2, 0.009~0.012 Hz. The 101 connectivity matrices for each subject were averaged to the 2 connectivity matrices according to the 2 frequency bands. If the ICN efficiency was considered in an uncertain low frequency (ULF) between LF1 and LF2, there was an ICN with a statistically significant difference in ULF from LF1 or LF2, but the tendency of ICN efficiency to frequency was maintained (See Supplementary Fig. S4). Thus, we analyzed contribution of ICN to brain network integration only in LF1 and LF2.

### Sparsity of the connectivity matrix

We constructed the connectivity matrices with 7 different sparsity levels (1%, 3%, 5%, 7%, 9%, 10% and 15%) because the sparsity level of connectivity matrix affects graph theoretic measures^54^. We decided upon a 5% sparsity level for further analysis because the sparsity levels surviving the minimum correlation value of 0.3 or greater were from 1% to 5% and the sparsity levels satisfying criteria for small-world properties [i.e. k_net_ > log(n)^55^] were from 5% to 15% (See Supplementary Fig. S5).

### ICN efficiency (E_ICN_)

We defined E_ICN_ to evaluate the contribution of ICN to brain network integration more directly. E_ICN_ was defined as

1 $${E}_{ICN}={E}_{Global}-{E}_{IC{N}^{{\rm{C}}}}\,$$

2 $${E}_{IC{N}^{{\rm{C}}}}=\frac{1}{{k}_{IC{N}^{{\rm{C}}}}}\sum _{i\in {K}_{IC{N}^{{\rm{C}}}}}\,{E}_{i,IC{N}^{{\rm{C}}}}=\frac{1}{{k}_{IC{N}^{{\rm{C}}}}}\,\sum _{i\in {K}_{IC{N}^{{\rm{C}}}}}\frac{{\sum }_{j\in {K}_{IC{N}^{{\rm{C}}}},j\ne i}{d}_{ij}^{-1}}{{k}_{IC{N}^{{\rm{C}}}}-1}\,$$

where, K_ICN_^C^ is the set of all nodes in whole brain network except ICN, k_ICN_^C^ is the number of K_ICN_^C^, and d_ij_ is the shortest path length between nodes i and j. E_ICN_ can detect the change of the shortest path length of the brain network when ICN was excluded and indicates the contribution of an individual ICN to brain network integration. Note that E_ICN_ cannot distinguish which region of the ICN affects the change of the contribution.

### Betweeneess centrality (BC) hub

We investigated the number of BC hubs as the indicator of E_ICN_ together. The BC measurement of nodes is the rate at which each node passes through the shortest path between other nodes^56^. BC was defined as

3 $$BC(i)=\frac{1}{(k-1)(k-2)}\sum _{\begin{array}{c}h,j\in K\\ h\ne j,h\ne i,j\ne i\end{array}}\frac{{\rho }_{hj}(i)}{{\rho }_{hj}}$$

where, K is the set of all nodes in the whole brain network, k is the number of K and ${\rho }_{hj}$ is the number of the shortest paths between h and j, and ${\rho }_{hj}(i)$ is the number of the shortest paths between h and j that pass through i. BC can detect the extent to which particular regions of an ICN involve the shortest path lengths within a brain network. BC hub was determined as the node that was one standard deviation above the network average BC^12^.

### Efficiency between each ICN and the other ICNs (E_ICN-ICN_)

We computed the E_ICN-ICN_ to investigate different integration with other ICNs of each sub-ICN along the frequency band. The E_ICN-ICN_ is the shortest path length between ICN and other ICNs. E_ICN-ICN_ was defined as

4 $${E}_{ICN-ICN}=\frac{1}{{k}_{IC{N}_{a}}\ast {k}_{IC{N}_{b}}}\sum _{i\in {K}_{IC{N}_{a}}}\,\sum _{j\in {K}_{IC{N}_{b}}}\,{d}_{ij}^{-1}\,$$

where, K_ICN_ is the set of nodes of ICN, k_ICN_ is the number of K_ICN_ and d_ij_ is the shortest path length between nodes i in ICN_a_ and j in ICN_b_. E_ICN-ICN_ can detect how many shortest paths each ICN has with other ICNs.

### Statistical evaluation

A paired sample t-test between the E_ICN_ of 2 frequency bands was performed to investigate whether the contributions of an ICN to brain network integration along the frequency bands were different from each other. The paired sample t-test of E_ICN_ was corrected by the significance level of p-value <0.005 with Bonferroni correction (Figs 2 and 5a). A paired sample t-test between the E_ICN_ of 2 frequency bands in sub-ICN was performed to investigate wheter the contributons of an ICN to brain network integration along the frequency bands were different from each other. The paired sample t-test of E_ICN_ in sub-ICN was corrected by the significance level of p-value <0.008 with Bonferroni correction (Figs 3 and 5b). Two sample t-test between the E_ICN-ICN_ of sub-ICN pair was performed to investigate the different integration with other ICNs along the frequency bands. The two sample t-test of E_ICN-ICN_ of sub-ICN was corrected by the significance level of p-value of 0.0025 with Bonferroni correction (Fig. 4).

## Supplementary information

Supplementary Table S1, Supplementary Figure S1-S5
