# Supplementary material for: Frequency specific contribution of intrinsic connectivity networks to the integration in brain networks
Source: Sci Rep. 2019 Mar 11;9:4072. doi: 10.1038/s41598-019-40699-z (PMC6412043; doi:10.1038/s41598-019-40699-z)

Frequency specific contribution of intrinsic connectivity networks to the integration in brain networks

Yeong-Hun Park[1], Jungho Cha[2], Viktoriya Bourakova[2], and Jong-Min Lee[1*]

[1]Department of Biomedical Engineering, Hanyang University, Seoul, Korea

[2]Memory and Aging Center, Department of Neurology, University of California, San Francisco, San Francisco, CA, USA

* *Corresponding author*

Jong-Min Lee

Dept. of Biomedical Engineering, Hanyang University

Sanhakgisulkwan # 319, 222 Wangsimni-ro, Seongdong-gu, Seoul, 04763, Korea

Tel. +82-2-2220-0685, E-Mail. ljm@hanyang.ac.kr

Supplementary Table S1

Supplementary Figure S1-S5

Supplementary Table S1. The difference of silhouette values between k=2 and other k-cluster solution about the number of clustering iteration.

The table shows the difference of silhouette values between k=2 (the highest silhouette value, Fig 1C) and other k-cluster solution for the number of clustering iterations. The significance level of p-value is 0.0002 (Bonferroni correction). The silhouette value of k=2 significantly differs from other k-cluster solutions after clustering 30 times.

K-cluster solution	K-cluster solution	P-value at 10 Times	P-value at 20 Times	P-value at 30 Times	P-value at 40 Times	P-value at 50 Times	P-value at 80 Times	P-value at 100 Times
2	3	0.0023	p < 2e-4	p < 2e-4	p < 2e-4	p < 2e-4	p < 2e-4	p < 2e-4
2	4	0.0010	p < 2e-4	p < 2e-4	p < 2e-4	p < 2e-4	p < 2e-4	p < 2e-4
2	5	0.0040	p < 2e-4	p < 2e-4	p < 2e-4	p < 2e-4	p < 2e-4	p < 2e-4
2	6	0.0120	0.0029	p < 2e-4	p < 2e-4	p < 2e-4	p < 2e-4	p < 2e-4
2	7	0.0441	0.0115	p < 2e-4	p < 2e-4	p < 2e-4	p < 2e-4	p < 2e-4
2	8	0.5190	0.2611	p < 2e-4	p < 2e-4	p < 2e-4	p < 2e-4	p < 2e-4
2	9	0.3570	0.0505	p < 2e-4	p < 2e-4	p < 2e-4	p < 2e-4	p < 2e-4
2	10	0.9533	0.3782	p < 2e-4	p < 2e-4	p < 2e-4	p < 2e-4	p < 2e-4
2	11	0.7850	0.2402	p < 2e-4	p < 2e-4	p < 2e-4	p < 2e-4	p < 2e-4
2	12	0.8543	0.2296	p < 2e-4	p < 2e-4	p < 2e-4	p < 2e-4	p < 2e-4
2	13	0.9105	0.1306	p < 2e-4	p < 2e-4	p < 2e-4	p < 2e-4	p < 2e-4
2	14	0.5196	0.0495	p < 2e-4	p < 2e-4	p < 2e-4	p < 2e-4	p < 2e-4
2	15	0.6501	0.0443	p < 2e-4	p < 2e-4	p < 2e-4	p < 2e-4	p < 2e-4
2	16	0.2425	0.0029	p < 2e-4	p < 2e-4	p < 2e-4	p < 2e-4	p < 2e-4
2	17	0.0808	0.0004	p < 2e-4	p < 2e-4	p < 2e-4	p < 2e-4	p < 2e-4
2	18	0.0873	0.0030	p < 2e-4	p < 2e-4	p < 2e-4	p < 2e-4	p < 2e-4
2	19	0.1191	0.0003	p < 2e-4	p < 2e-4	p < 2e-4	p < 2e-4	p < 2e-4
2	20	0.0172	p < 2e-4	p < 2e-4	p < 2e-4	p < 2e-4	p < 2e-4	p < 2e-4

Supplementary Figure S1. The results of silhouette value according to the number of clustering iterations.

When we repeated the clustering from 10 to 100, the silhouette values always have the highest value when k=2.

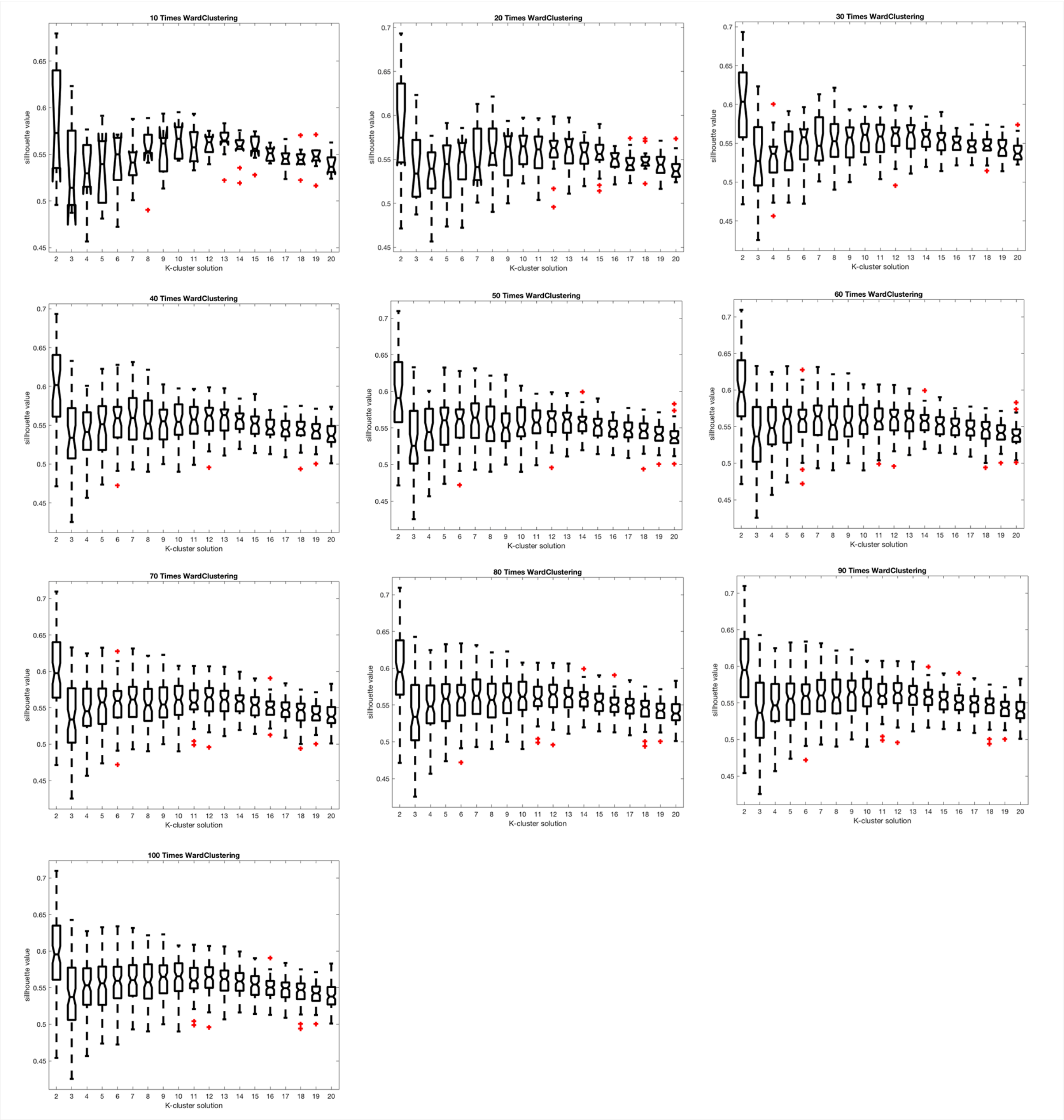

Supplementary Figure S2. Impact of preprocessing pipeline on E_{ICN} **by frequency.**

Difference of E_{ICN} by frequency according to the preprocessing pipeline. The E_{ICN} results which included the volume censoring step differed from the the E_{ICN} results which included only the ICA-fixed step in statistical significance of AN, VN, LN, ECN and PCN, but the tendency of E_{ICN} to frequency was maintained. The E_{ICN} results which included the GSR and volume censoring step differed from the E_{ICN} results which included the volume censoring step in statistical significance of VN, but the tendency of E_{ICN} to frequency was maintained.

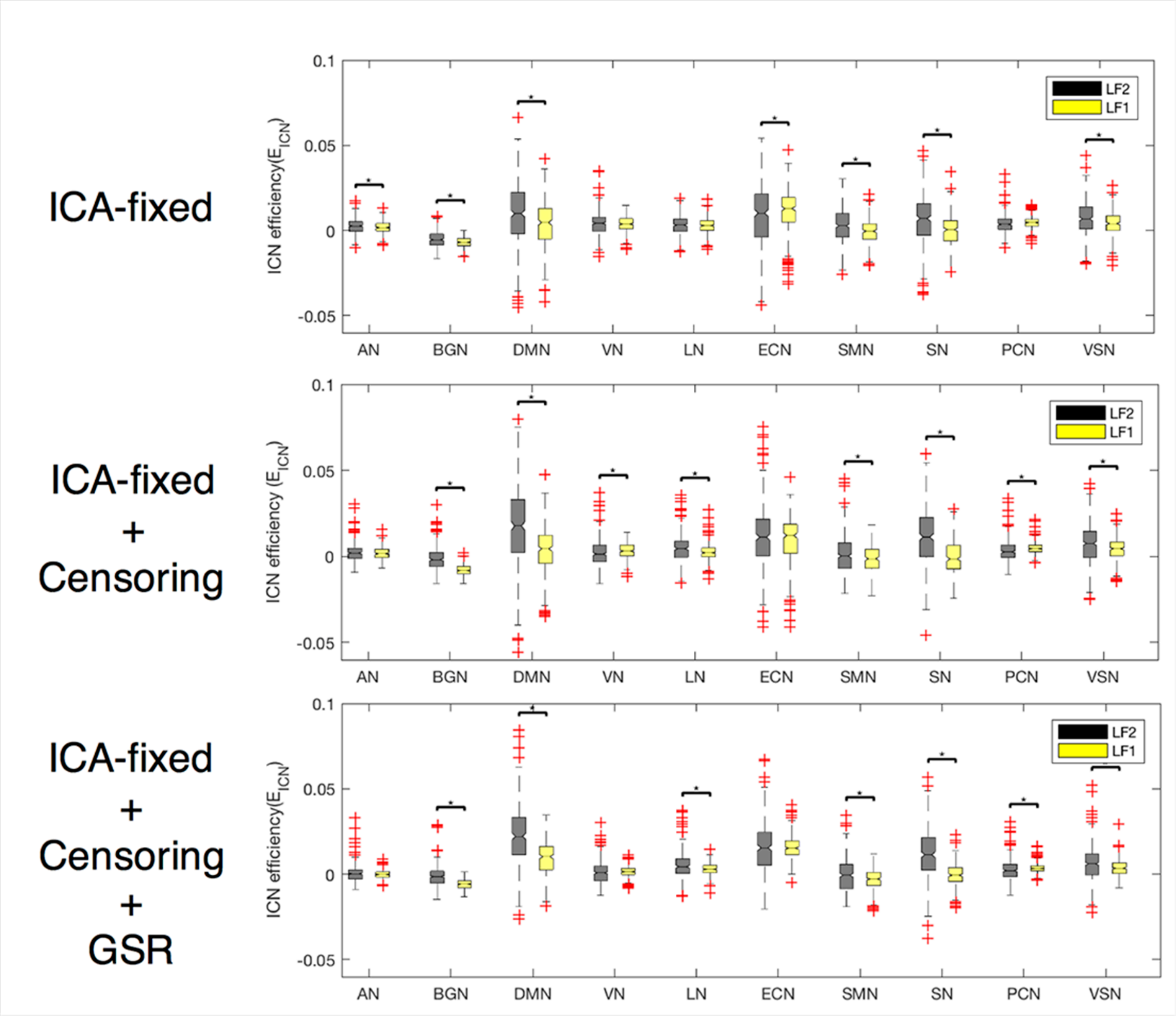

Supplementary Figure S3. Results of cluster solution in several hyperparameter condition.

In the any conditions, cluster solution 2 had the hightest silhouette value, so we used s_0=1 and ds =1.

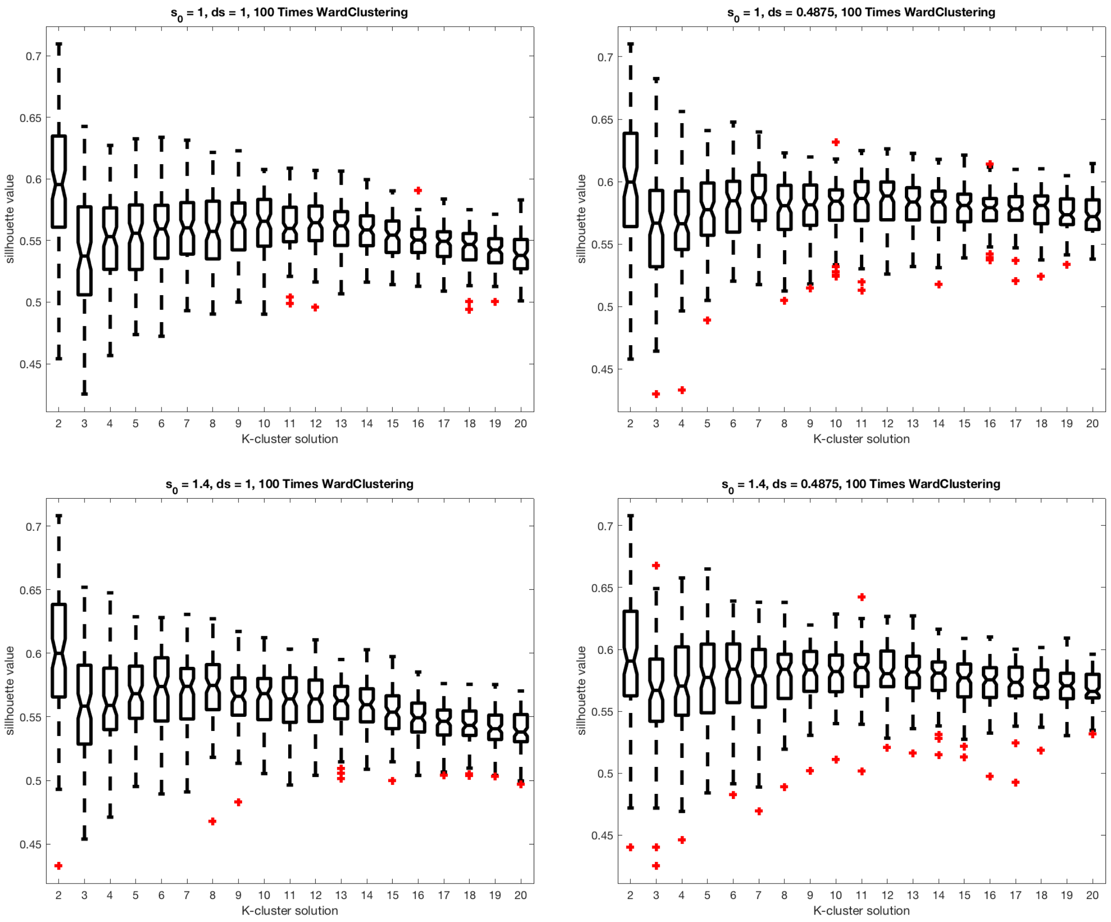

Supplementary Figure S4. The E_{ICN} along the frequency band including uncertain low frequency (ULF)

If the E_{ICN} is considered in an ULF (0.012 ~ 0.03 Hz) between LF1 and LF2, there is an ICN with a statistically significant difference in ULF from LF1 or LF2, but the tendency of E_{ICN} to frequency is maintained. Analyis of variance (ANOVA) was performed for each ICN to determine the difference in E_{ICN} with frequency ($p < 0.005$ with Bonferroni correction). *Post hoc* two-sample t-tests for differences between the E_{ICN} of each frequency band were also performed ($p <0.01$ with Bonferroni correction).

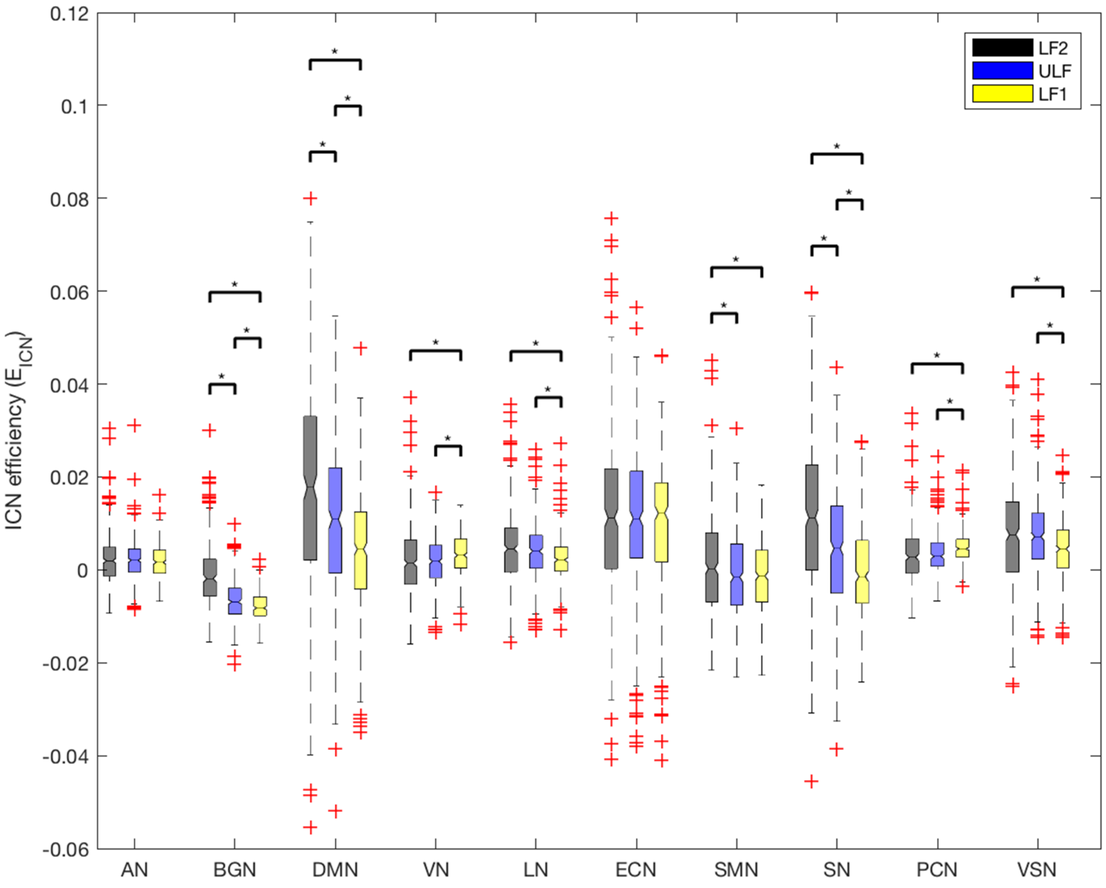

Supplementary Figure S5. The characteristics of the connectivity matrix according to sparsity. (a) Mean degree of connectivity matrix at the sparsity for each frequency. The threshold is the logarithm of the number of whole brain network nodes. At a sparsity of 5% or more, the mean degree exceeds the threshold and satisfies the small-worldness network. (b) The surviving correlation coefficient at the sparsity for each frequency. The mean of histogram for each subject's surviving correlation coefficients are plotted for each case. There were higher surviving correlation coefficient values at LF2 than at LF1. Sparsity at 5% is satisfied at a correlation coefficient of at least 0.3. The top row indicates results at LF1. The bottom row indicates results at LF2.

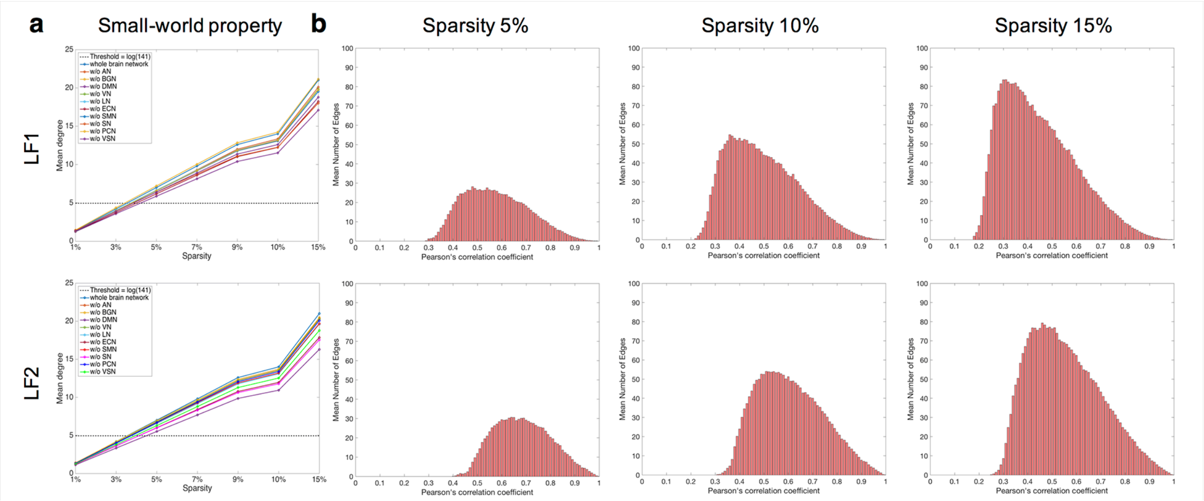

Supplement: Supplementary file 1 — Supplementary Table S1, Supplementary Figure S1-S5 [file 41598_2019_40699_MOESM1_ESM.pdf]
